# A report of a rare congenital malformation in a Nepalese child with congenital pouch colon: a case report

**DOI:** 10.1186/1757-1626-2-6424

**Published:** 2009-03-10

**Authors:** Vikal Chandra Shakya, Chandra Shekhar Agrawal, Rabin Koirala, Sudeep Khaniya, Prakash Poudel, Shailesh Adhikary

**Affiliations:** 1 Department of Surgery, B P Koirala Institute of Health Sciences, Dharan, P.O. Box 7053, Kathmandu, Nepal; 2 Department of Pediatrics, B P Koirala Institute of Health Sciences, Dharan, P.O. Box 7053, Kathmandu, Nepal

## Abstract

Congenital pouch colon is one of rare congenital anomalies. We report a 3-day-old male child with congenital pouch colon who underwent a window colostomy but died because of overwhelming sepsis. Due to its rarity, many surgeons in our part of the world may not be aware of it, hence increasing the potential to its mismanagement. However, with simple keen observations, we can safely come to its diagnosis. The aim of this report is to bring attention to congenital pouch colon associated with anorectal malformation in our country, with a brief emphasis on an approach to its diagnosis and initial management.

## Introduction

Congenital pouch colon is a rare anorectal malformation in which the distal bowel is shortened forming a pouch-like dilatation, the pouch usually terminating in a fistula communicating with the genitourinary tract. There is a wide anatomical variation, ranging from a complete absence of normal colon with the distal ileum opening into the colonic pouch, to the presence of a near complete length of normal proximal colon, with only the rectum or rectosigmoid colon being affected. CPC has been described in very few literatures, mostly from India [1]. Here we report a case of congenital pouch colon who presented in our hospital, it is first of its kind reported from our country and we endeavor to discuss briefly its diagnosis and its early management.

## Case Presentation

**Figure 1:**
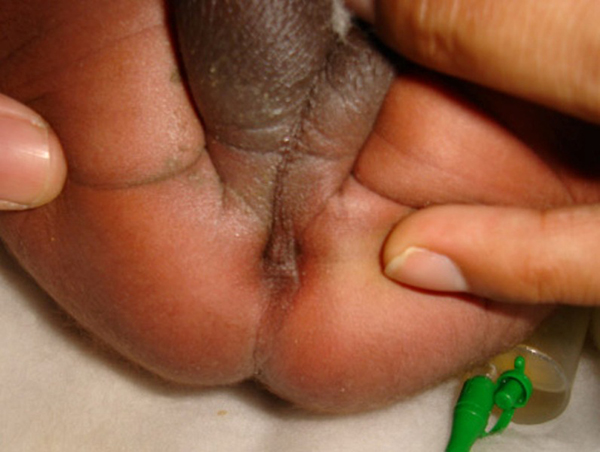
**Perineum of the child showing no anal opening**.

**Figure 2:**
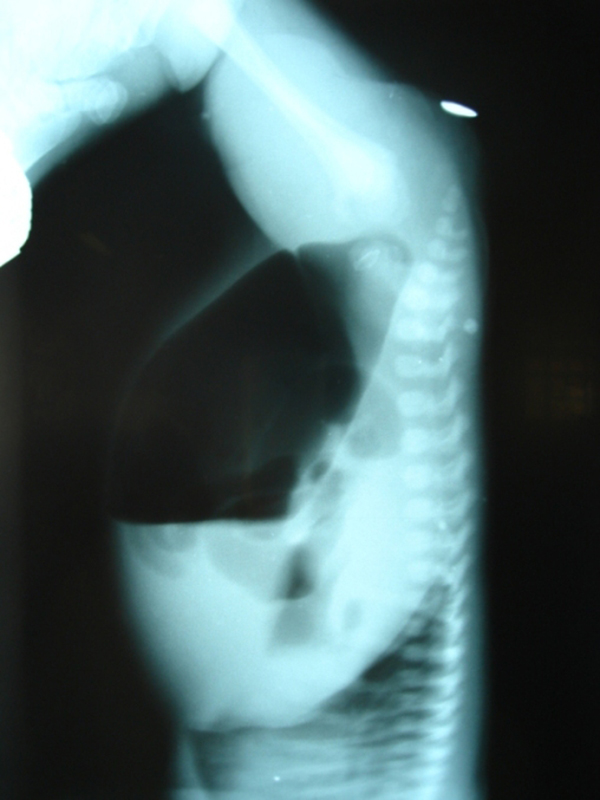
**Invertogram of the child showing high type of ARM**.

**Figure 3:**
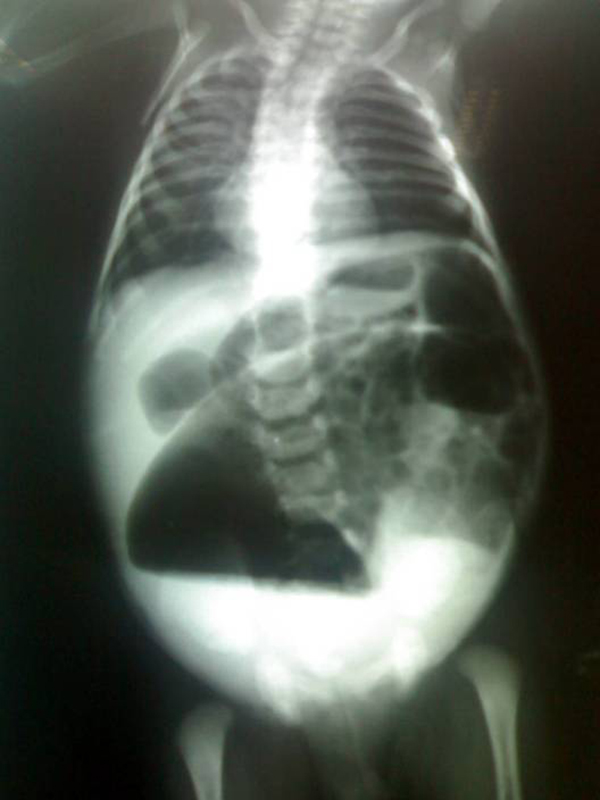
**An erect abdominal radiograph showing a dilated bowel loop with an air fluid level occupying more than half of the diameter of the abdomen**.

**Figure 4:**
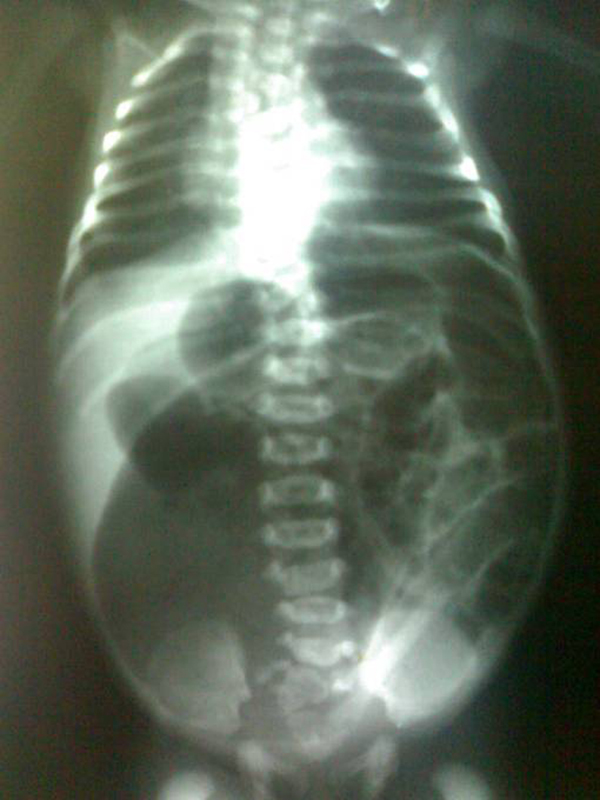
**A supine abdominal radiograph showing large dilated bowel loop occupying more than half of the diameter of the abdomen**.

**Figure 5:**
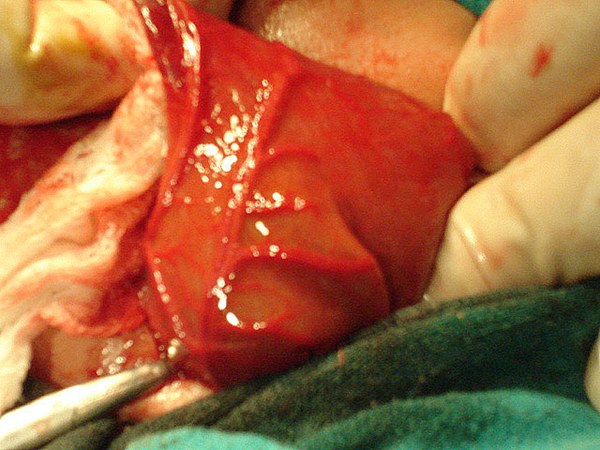
**Laparotomy showing the dilated sigmoid colon forming a pouch**.

A 3-day-old male child, delivered vaginally at term, was brought to our hospital with complaints of failing to pass stool since birth and abdominal distension. There was no history of meconuria. On examination, his weight was 2.5 kg, was dehydrated, lethargic, had gross abdominal distension, and was found to have no anal opening (Figure 1). A nasogastric tube was placed to rule out associated tracheoesophageal fistula. An invertogram was done which showed high type of anorectal malformation (Figure 2) and an abdominal radiograph done in an erect and supine position showed a large bowel loop and an air-fluid level dilated to more than half of the transverse diameter of the abdomen (Figure 3,4). A diagnosis of congenital pouch colon was made and the child was planned for exploration. On laparotomy, the sigmoid was found to be largely dilated to form a pouch (type 4 CPC) (Figure 5) and a window colostomy was made out of the dilated sigmoid. The stoma started to function on the first postoperative day; however the child died on the second day possibly due to sepsis.

## Discussion

Congenital pouch colon (CPC) was first reported by Trusler et al in 1954 [2]. It has mostly been reported from India, few cases from other countries like China, Japan, UK, USA, Sweden, and Saudi Arabia [1,3-6]. It is more common in males (up to 3.5:1) [4,7]. There are wide anatomical variations of CPC. The classification proposed by Narasimharao et al is based on the length of normal colon proximal to the colonic pouch [8]. In type 1, the ileum opens directly into a pouch; in type 2, the ileum opens into a short segment of the cecum, which then opens into a pouch; and in type 3, at least 10 to 15 cm of normal colon is present between the ileum and the pouch, in type 4, only the terminal portion of the colon (sigmoid or rectum) is converted into a pouch.

CPC is a rare variant of ARM, and it is distinctly different from other ARMs. Preoperative diagnosis of CPC requires a high degree of suspicion, especially to pick up the dilated terminal bowel loop and an air-fluid level that occupies more than half of the diameter of the abdomen on an abdominal radiograph, which is so nearly forgotten in evaluating a child of imperforate anus.

There are different options in the management of CPC [9]. It ranges from a window colostomy to division of colourinary fistula, excision of pouch, and an end colostomy through normal colon; coloplasty and end colostomy; proximal colostomy to a single stage pullthrough [10-12]. In most early reports, a window colostomy of the pouch was the most frequently performed procedure at initial presentation [2,7]. It is the simplest surgery and can be done in a minimum time period. However, window colostomy is associated with lots of complication such as poor functioning and incomplete decompression of pouch, recurrent urinary tract infection due to persistent colourinary fistula and associated vesicoureteric reflux, stomal stenosis, massive prolapse often with eversion of the entire colonic wall and excoriation [1,9]. The mortality following window colostomy has been reported to be in the range of 15-20% [11]. At present, this short and simple procedure still has a role as the initial surgery in the sick neonate. It was contemplated in our case also in view of the delayed presentation and poor general condition. The probable cause of death in our child was sepsis due to urinary tract infection secondary to a persistent colourinary fistula. Due to these reasons, the preferred procedure is excision of the pouch, ligation of colourinary fistula with an end colostomy [3]. It has also been shown to be associated with maximal survival [13]. The single stage surgery for CPC though described has been found to have high rate of complications and hence not recommended at present [3,9,12].

Etiology of pouch colon points to an early vascular insult in the form of intra-uterine obliteration of inferior mesenteric artery [14]. Clustering of cases in India has been believed to be associated with environmental factors such as deficiency of iodine and vitamin B, low socio-economic status and the extensive use of pesticides in the farm. These are postulated to disturb the embryogenesis when the hindgut is developing and differentiating into urinary and intestinal tracts [9]. These factors probably play a role in our country as well. A high index of suspicion, early treatment, and awareness of different options on management may lead to improved survival.

## Abbreviations

CPC: Congenital pouch colon; ARM: Anorectal malformation.

## Consent

Written informed consent was obtained from the patient's parents for publication of this case report and accompanying image. A copy of the written consent is available for review by the Editor-in-Chief of this journal.

## Competing interests

The authors declare that they have no competing interests.

## Authors' contributions

VCS, SK and PP made substantial contributions to concept and design of the article and acquisition of materials. CSA, RK and SA contributed significantly in critical revision and drafting the manuscript. All authors read and approved the final version of the manuscript.
